# A compact automated microprocessor–based stopped–flow analyser

**DOI:** 10.1155/S1463924680000260

**Published:** 1980

**Authors:** Michael A. Koupparis, Ken M. Walczak, Howard V. Malmstadt

**Affiliations:** 1 School of Chemical Sciences University of Illinois at Urbana-Champaign Urbana Illinois 61801 USA; 2 Laboratory of Analytical Chemistry University of Athens Greece Greece


### A compact automated microprocessor-based stopped-flow analyser

**Michael A. Koupparis*, Ken M. Walczak and Howard V. Malmstadt†**
*School of Chemical Sciences, University of Illinois at Urbana-Champaign, Urbana, Illinois 61801, USA.*

### Introduction

The stopped-flow technique for the rapid mixing of chemical reagents has been most frequently used to obtain fundamental information about rapid chemical reactions (rate law information, rate constants, activation energies, etc). It was also demonstrated, about a decade ago [1-3], that the stopped-flow technique is extremely useful for routine high-speed analytical/clinical quantitative chemical determinations. The stopped-flow sampling and mixing system can provide analytical information in a very short time, in a few seconds or even less than a second [1]. More recently, [3,4] it was shown that only a small sample and reagent volumes are required and the technique can be used for either reaction-rate or equilibrium methods.

A minicomputer-based stopped-flow system with automated control, data acquisition, data processing, and solution preparation has led to significant increases in measurement throughput, precision, accuracy and flexibility [5]. A much simpler, inexpensive and very compact stopped-flow system which is optimized for routine applications is described in this paper. The entire system is automated using a Rockwell AIM 65 microcomputer with self-contained keyboard, display, printer and BASIC ROMS for control of all operations, communication between modules, data acquisition and reduction, display and printout of results. The typewriter-size microcomputer and equally compact sample-handling and photometric analyser modules occupy only about one-half square meter of bench space. The programs are provided for specific applications utilising either reaction-rate, kinetic, or equilibrium quantitative analytical procedures. The characteristics of these programs and analytical data are presented for the determinations of ascorbic acid, iron, phosphorus, creatinine, and glucose. These data demonstrate the high precision, accuracy, and flexibility of this simple, compact and relatively inexpensive microprocessor-based analyser.

The sampling-mixing system is based on an automatic dual pipetter/mixer developed in the authors' laboratory that provides aliquoting, transferring, and mixing of solutions with a precision better than 0.1% [6]. The system requires no operator attention during normal use. All control functions are readily implemented and are TTL-compatible. The photometric system is an easily constructed module with a 1 cm flow cell, automatic shutter, light source, photomultiplier, and interference filters. After the initialisation the integration time is selected, the dark current and 100% transmittance are measured, and an investigative step allows the operator to find the appropriate delay time and measurement time for the optimum results. A routine program then allows the repetitive determination of a specific constituent in standard and sample solutions placed on a turntable. Rate measurements can be made, after a selected delay time, on time scales from 1.5 seconds to several minutes using linear regression on the absorbance. An endpoint or equilibrium measurement is made by a selected number of integrations of photomultiplier current. The absolute absorbance value is determined from this data after a selected delay time.

The operator interacts with the system through the keyboard, a 20 character display, and the 20 column thermal printer. In addition two cassette tape recorders can be used for storing programs or data.

### Instrumentation

A diagram of the system configuration is shown in Figure 1. In operation the standard or sample solutions are drawn into the stopped-flow unit from the turntable and the reagent from a reservoir, then the two solutions are forced through a mixer into the observation cell. The output current of the photomultiplier tube is converted to a proportional frequency by performing a current-to-voltage conversion followed by a voltage-to-frequency conversion. This signal is integrated for a fixed period of time by the microcomputer, and the resulting count stored in memory. At the end of a data acquisition cycle, the data are processed and the results are printed.

### Reagent/sample handling system

The system used is similar to one previously described [6]. The two syringes are driven by a double-acting air cylinder (Mosier Industries, Inc. model TSR, Brookville, Ohio) with a 0.75/in bore and a 2 in stroke. The cylinder is controlled by two subminiature solenoid valves (Model 3NO686, Angar Scientific Corp., East Hanover, NJ 07936). The air cylinder movement is coupled to the syringe plungers by the plunger block which allows both syringe plungers to move during the filling and delivery processes. The length of the stroke for the air cylinder may be varied by adjusting a mechanical stop to adjust the volume delivered. (A brass screw 5/8 in diameter with a cover of TFE to minimize noise during the metal collision is used.)

Miniature air regulators and gauges (models RO4-100-RNK AV & 18-013-237 Walter Norris Engineering Co., Chicago, IL 60648) are used to provide a pressure of 18 psi and 24 psi, the latter for delivering the solution aliquots. The former is used for filling the syringes. The 18 psi fill pressure allows for a less than one second aliquot step without degassing of the solutions. The 24 psi is optimised for adequate delivery pressure with efficient mixing of the solutions as they pass through the X-Y mixer. Two commercially available, 3-way valves were used in the unit for automatic control of the solution flow. They comprise an inlet port for tubing which is connected to the sample and reagent reservoirs, a syringe port in which the syringe assembly is mounted and an outlet port for the solution to be delivered from the syringes. A KEL-F slider is pneumatically positioned in each valve to interconnect the desired ports by a pneumatic actuator (Laboratory Data Control, Model PA-875K, Riviera Beach, Fl), operated by the

*Present Address: Laboratory of Analytical Chemistry, University of Athens Greece
†Address correspondence to this author.

subminiature solenoid valve described previously and using 75 psi air pressure. A spring return (Model SR1K) returns the slider to its normal rest position (filling position). A small screw is added to the end of the spring return to monitor the valve position (activated or deactivated) in conjunction with an optical interrupter module (General Electric H13B2). Coupling with the feedback in the control circuitry ensures that the delivery of the aliquoted solutions does not begin until the pneumatic actuator has moved the sliders into the delivery position.

These valves have an internal volume of only 42μl. The low dead volumes are useful for applications requiring expensive reagents and for rapid changeover of samples. A KEL-F valve body is used because it is inert to the strongly acidic solutions required in some applications. A less expensive nylon body can also be used when the inertness of KEL-F is not necessary. The bodies of the valves were used as provided by the manufacturer. The sliders are modified (a) so that the solutions are aliquoted through the top of the valves and into the syringes and (b) that the delivery step takes place by passing the solution straight through the valve into the X-Y mixer. These modifications were achieved by plugging the vertical channel in the delivery position and extending the horizontal channel completely through the slider.

A check valve (Lee – Instac model TCKA6201050A) placed after the flow cell output, provides a back pressure during the delivery operation and avoids the generation of fine bubbles. This cavitation can cause a sharp decrease in the light transmittance by the solution in the observation cell. This inexpensive check valve can replace other, more expensive, back pressure systems in many applications.

**Mixer**

The X-Y mixing chamber is constructed from two pieces of PTFE containing a X and Y channel for mixing the solutions. The channel diameters in the mixer are 0.8 mm to be compatible with the valve and the interconnecting tubing used in the unit. The PTFE was chosen because it is inert to strong alkaline and acidic solutions used in applications described here. It also forms a good seal between the interface of the two halves of the mixer when they are clamped together with two aluminium plates. The ports connecting the mixer and valve and the measurement cuvette are constructed to accept the commercial ¼ in (28 tpi) tube end fittings which are used throughout the unit. Connections are made with the observation cell and valves using PTFE tubing of 0.8 mm inside diameter. The X-Y mixing configuration provides good mixing of solutions and a low dead volume (53μl).

**Photometer**

A tungsten light source (Heath model EU-701-90) with a regulated tungsten lamp power supply (Heath model EU-44A) is used. A deuterium light source (Heath model EU-701-91) replaces this source for work in the ultraviolet region. A series of 3-cavity, 25 mm diameter interference filters (Ditric Optics, Inc. Marlboro, MA 01752) are used for wavelength isolation. A lens placed between the light source and the shutter focusses the light beam on the observation cell. An 18μl flow cell (Hellma model 178.12) with 10 mm light path is used. A photomultiplier module (Heath EU 701-93) with a high voltage power supply (Model EU 42-A) is used as the detector. The automatic shutter is shown in Figure 2. A Guardian solenoid (24 VDC) moves a

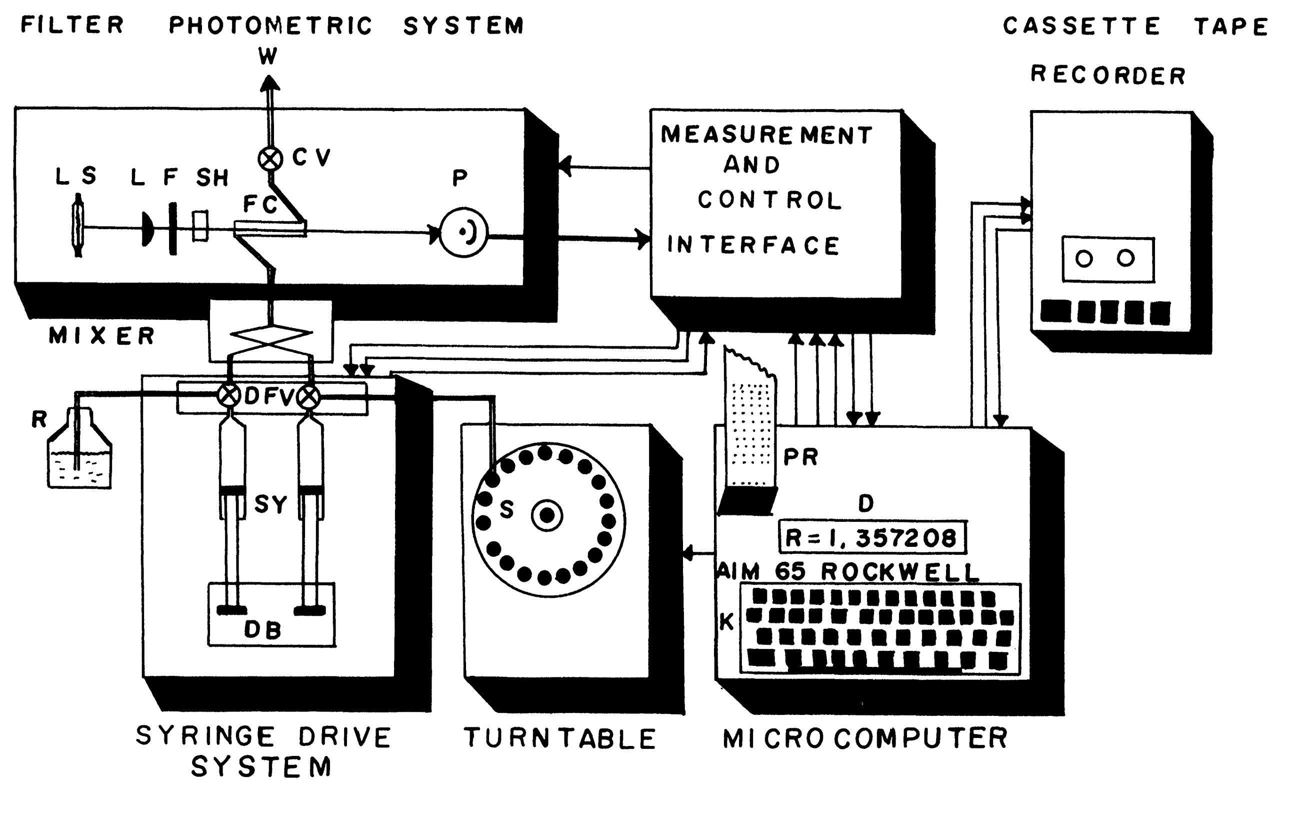

*Figure 1. General block diagram of the system. LS=Light Source, L=Lens, F=Filter, SH=Shutter, FC=Flow Cell, CV=Check Value, W=Waste, P=Photomultiplier, R=Reagent, DFV=Dual 3-way Flow Valves, DB=Drive Block, SY=Syringes, S=Samples, PR=Printer, D=Display, K=Keyboard.*

brass rod with a 2 mm aperture to open and close the light beam. The power supply and the control circuit for the automatic shutter are shown in Figure 5.

### Microcomputer system

The Rockwell AIM 65 (Rockwell International, Anaheim, CA) microcomputer has 4K of random-access memory, 8K BASIC, a 4K assembler, and an 8K monitor which are held in read-only memory. A 20-character visual display and a thermal printer (5x7 dot matrix, 120 lines per minute) of 20 character length are also features of this computer. The display may be used independently of the printer to prompt the operator at various points in the experimental sequence. The printer is used only when hard copy is desired.

Input/output operations are performed through an on-board peripheral chip, the R6522 Versatile Interface Adapter (VIA). One part of this chip contains two 8-bit parallel I/O ports (port A and port B). Each bit can be individually selected to be either an input or an output. For this application, the A port is used for input flags. The B port is used to output control signals and to input pulses from the voltage-to-frequency converter. The peripheral chip also contains two user-programmable 16-bit timers which generate or count pulses. Normally, in a data acquisition interface based on a voltage-to-frequency conversion, external circuitry must be added in the form of a real-time clock and a series of counters and latches to provide the complete interface. In this application, the peripheral chip's timer 1 functions as a time base and timer 2 as a counter foı the voltage-to-frequency converter output.

The manner in which the I/O ports and timers operate is determined by the content of three registers, the data direction registers of ports A and B and the auxiliary control register. Table 1 is a listing of the locations of these registers, the contents of which are loaded in the registers in this application, and their resulting function. Loading a 1 in any bit of a data direction register designates that bit as output while a O results in a bit being an input. Port A is used for input (DDRA = $ OO) and all of port B, except PB6, for output (DDRB = $ BF). (Note: $ indicates hexidecimal notation.) The operation of the timers is programmed by specific codes assigned to bits 5-7 of the auxiliary control register. Timer 1 is set in its free-running mode and timer 2 is set to count

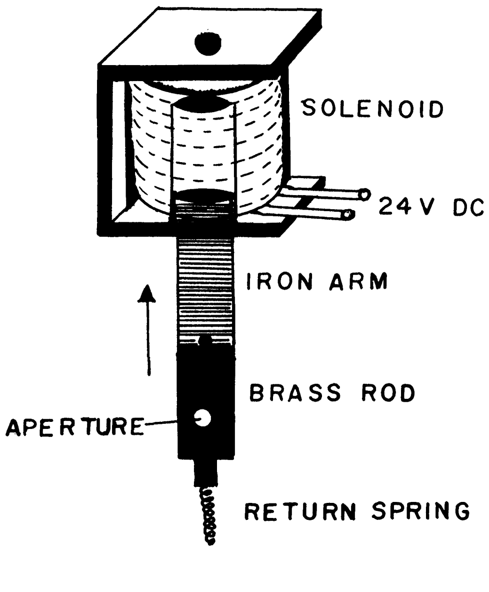

*Figure 2. Automatic shutter.*

**Table 1. Memory assignments and functions of control registers**

| Location* | Contents* | Register | Programmed function |
|---|---|---|---|
| A002 | BF | Port B Data Direction Register (DDRB) | Sets all bits as output except PB6 |
| A003 | 00 | Port A Data Direction Register (DDRA) | Sets all bits as inputs |
| A00B | 60 | Auxiliary Control Register (ACR) | Timer 1 free-running mode, generates continuous interrupts, output to PB7 disabled. Timer 2 counts pulses input at PB6 |

*Hexidemical notation.

negative going pulses occurring at PB6 (ACR = $ 60). These registers are loaded with their assigned values in a machine language subroutine that is called at the beginning of any program.

Each timer consists of one or two 8-bit latches and a 16-bit down counter. In the normal operation, the latches are loaded by the program with a desired number. The completion of the write operation clears the timer's corresponding flag in the interrupt flag register, loads the counter with the value in the latches, and begins to decrement the value in the counter with each pulse at its input. These pulses normally are input from the phase two clock of the microcomputer. This 1 MHz clock arises from a 4 MHz crystal oscillator of the microcomputer and provides an accurate time base. One of the modes of timer 2 enables it to count negative-going pulses occurring at bit 6 of port B. These pulses must be $> 2\mu s$ in pulse width and pulse spacing. When the counter is decremented to zero, the interrupt flag associated with that timer is set.

Programs are written in BASIC as a series of subroutines which perform selected tasks. To perform an analysis, these subroutines are called in the desired order to fulfill the requirements of the analytical method. Separate machine language subroutines called by BASIC are used to perform selected functions. Machine language subroutines are written to initialise the I/O ports and timers, to provide a programmable delay period, and to acquire data.

Machine language routines are called from the BASIC program by loading their starting address at $ 04 (LSB) and $ 05 (MSB) using the POKE command and then executing BASIC's USR function. When initially setting up BASIC's memory parameters, sufficient memory must be allocated for the machine language routines by limiting the memory assigned to BASIC.

A programmable delay time is produced by counting interrupts generated by timer 1 using a machine language subroutine. The flow chart and program for this subroutine is shown in Figure 3. First, the length of delay (DT) desired in hundredths of seconds is passed from the BASIC to the subroutine through the USR function. It is stored as a 16-bit number in locations $AC and $AD by calling a subroutine in the BASIC ROM. Timer 1 is then calibrated to generate interrupts approximately every 0.01 second by loading $0E into the lower latch of timer 1 ($AOO6) and $27 into the higher latch of timer 1 ($AOO5). This operation causes the timer to begin counting the microcomputer's phase two clock pulses, setting bit 6 of the interrupt flag register when the counter reaches zero. The program waits for the timer 1 interrupt to occur, then clears the flag, and substracts one from the DT value in $ AC and $ AD. If $ AC and $ AD have not decremented to zero, the program returns to await the next interrupt from the timer. Once DT has been decremen-

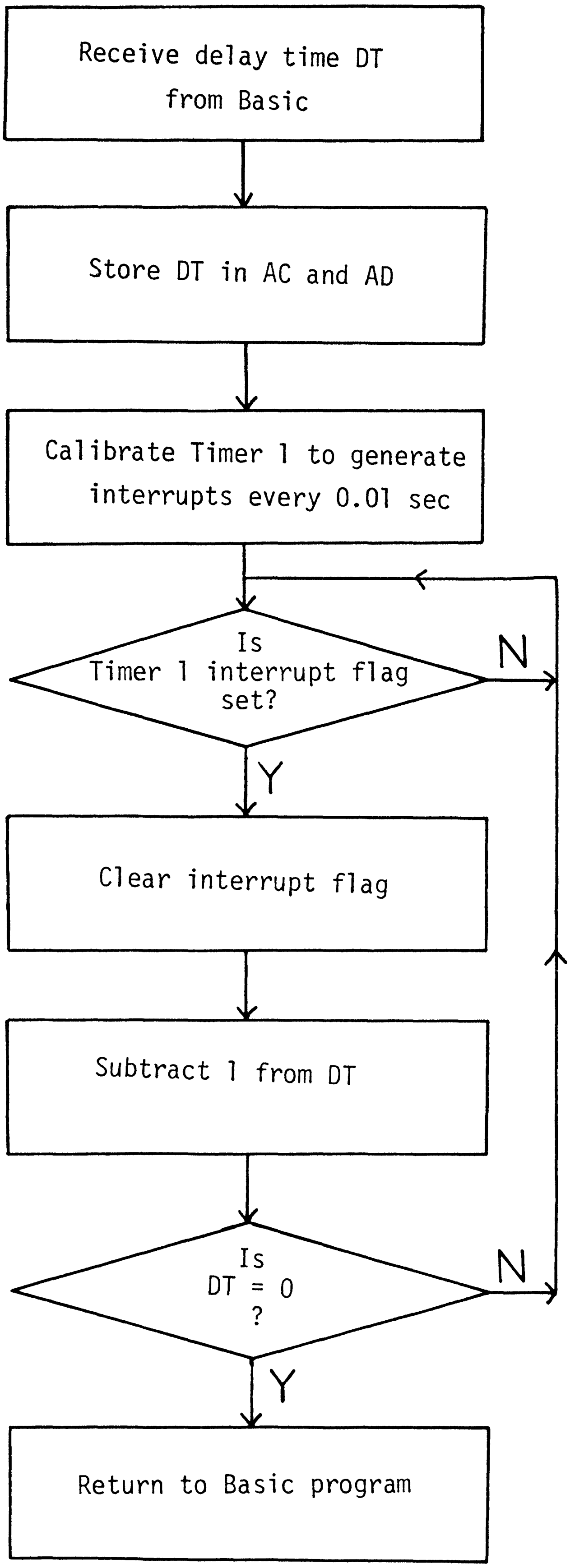

*Figure 3(a)*

ted to zero, the program returns to BASIC by jumping to a routine at $ COD1. This subroutine is used to provide tne delays to control the pneumatic valves and for delays needed to measure the absorbance.

The data acquisition subroutine counts V–F pulses with timer 2 for a set integration time IT measured by timer 1. The flowchart and program for this subroutine are shown in Figure 4. Timer 1 performs in an identical manner as in the delay subroutine. The integration time IT is passed to the subroutine from BASIC using the USR function. This 16-bit value is stored in two locations designated HILP and LOLP. Timer 1 is then calibrated to generate interrupts every 0.01 seconds. Timer 2 is loaded with its maximum value $ FFFF. This begins the counting operation, with the timer 2 counter value being decremented with each pulse occurring at PB6. The program then waits for an interrupt from either timer. The timer 1 interrupt flag is then checked to see if it caused the interrupt. If the result of this check is negative, the interrupt must be due to timer 2 decrementing to zero. In this case, a jump instruction is given to a routine, and an overflow message is printed. From this, the operator knows the photomultiplier voltage or the integration time must be decreased.

If the interrupt is due to the timer 1 flag, the flag is cleared and then the contents of timer 2's counter are immediately read and stored in locations $ OE75 (T2HI) and $ OE76 (T2LO). The value IT is then decremented by one and checked to see if the integration time is over. If not, the program returns to wait for the next interrupt. When IT is decremented to zero, the number of pulses counted during the integration time is calculated using the values stored in T2HI and T2LO. This value is then returned to BASIC as a 16-bit floating-point number by jumping to a routine in ROM at $COD1.

Software has been written to provide several modes of operation. These include the investigative mode, the interactive routine mode, and the dedicated mode. Quantitative chemical determinations can be made using equilibrium or reaction-rate methods.

```
A    MC MIM OP
0EA7 20 JSR BEFE
0EAA A9 LDA #0E
0EAC 8D STA A006
0EAF A9 LDA #27
0EB1 8D STA A005
0EB4 A9 LDA #40
0EB6 2C BIT A00D
0EB9 F0 BEQ 0EB4
0EBB AD LDA A004
0EBE 38 SEC
0EBF A5 LDA AD
0EC1 E9 SBC #01
0EC3 85 STA AD
0EC5 A5 LDA AC
0EC7 E9 SBC #00
0EC9 85 STA AC
0ECB A9 LDA #00
0ECD 05 ORA AD
0ECF 05 ORA AC
0ED1 D0 BNE 0EB4
0ED3 4C JMP C0D1
```

*Figure 3(b)*

*Figure 3. Flowchart and program for delay time machine language subroutine. A = Memory Address (Hex), MC = Machine Code, MIM = Machine Instruction Mnemonics, OP = Operand.*

The investigative program is an interactive program in which the operator can vary the measurement parameters of delay and measurement time. This mode is used to develop a method and to optimise the time period at which a measurement is made. This is especially important for reaction-rate methods.

The interactive routine program is capable of running a series of standards and samples and further developing a methodology. This mode gives the operator the capability of choosing the measurement parameters as in the investigative mode but does not offer the option of changing these once they have been entered. With this program a series of standards can be run and a calibration curve is computed. This can be used to quantitate an analyte in samples run later. The operator interacts with this program through a series of prompts on the display. Statistical data on every determination is calculated and recorded on the printer.

The power of the computer is used to its greatest extent in the dedicated mode of operation. In this mode, experimental parameters previously determined for a specific assay are included in the program. Though the basic operation is identical to that of the interactive routine mode, virtually all operator interaction with the computer is avoided once the number of samples to be assayed is typed in. The program initialises the system, sequences through the standards and samples on the turntable, and computes and prints the statistical data on the calibration curve. Statistical information and the analyte concentration in each sample are also computed using this mode. Error checks are provided throughout the program sequence both on the mechanical operation of the instrument and the validity of the measurements. If an error occurs, a message informs the operator of the problem and indicates its probable cause.

### System interface

A diagram of the system interface is shown in Figure 5. This consists of a measurement interface, a control interface for the reagent-sample handling system, and the circuitry to control the automatic shutter.

The measurement interface consists of two operational amplifiers. One acts as a current-to-voltage converter and the other as an inverter amplifier which makes the output compatible with the voltage-to-frequency converter. The current from the photomultiplier tube is fed to this circuit

*Figure 4. Flowchart and program for data acquisition machine language subroutine. A=Memory Address (Hex), MC=Machine Code, MIM=Machine Instruction Mnemonics, OP=Operand.*

and this results in a signal at the output which varies from O to −10V. This signal is input to a National Semiconductor LM331 voltage-to-frequency converter in its precision configuration [7] (100kHz full scale, ± 0.03% non linearity, ± 50 ppm/°C maximum). The pulsed output of the converter is fed to pin 6 of port B and is counted for a fixed time by the 16-bit timer 2 of the microcomputer.

The control interface has been described previously [5]. The two control signals, *fill* and *fire*, are connected to PBO and PB1 outputs of port B. An opto-interrupter circuit is used to provide an end-of-push signal connected to pin 7 of port A. This provides a timing flag from which the beginning of a data acquisition cycle is referenced.

The interface that controls the automatic shutter is connected to the PB4 output of port B. It uses an optical isolator that enables a transistor to activate the pulling solenoid. Activation of the solenoid moves an aperture from its normal position, interrupting the light path.

The control and shutter circuits are activated by a logic 1 to 0 transistion from the microcomputer. The 1 to 0 transition was chosen as the power-on state of the parallel I/O ports to ensure that the control signals were not activated to the microcomputer or a reset sequence was initiated. Although these signals could be connected directly to the computer, as a precaution all signals are buffered.

### Systems operation

To operate the automated stopped-flow instrument the desired software package is first loaded from the cassette recorder into the computer's memory. Since programs in each of the three modes follow a similar progression, the interactive routine mode will be used as an example of the system operation. The flowchart for a typical sequence in a determination using this program is shown in Figure 6.

Initially BASIC assigns values to several of the more commonly used variables in the program. A subroutine, in machine code, is then called to set up the I/O ports and the two timers.

Once the peripheral chip is programmed, the operator is prompted by the display as to whether the blank is ready to be injected into the flow cell and how many flushes of the system are desired. When this response is typed, the program checks whether the syringe drive mechanism is in its *fill* or *fire* position, and then proceeds to the *fill* and *fire* sequence for the designated number of times, ending in the *fire* position.

The integration time desited for the measurement is then set by the operator. This can be varied from a millisecond to periods of over five minutes. Once this is set, the program passes into the machine code pulse counting routine which continuously updates the count obtained during each integration period in real time and displays it in hexadecimal notation using monitor routines provided by the microcomputer. With this feature, the photomultiplier tube voltage can be adjusted to maximise the count obtained from the voltage-to-frequency converter. Once this is done, the operator hits the escape key and is asked whether the parameters are satisfactory.

A positive response results in closing of the automatic shutter and the dark count is obtained followed by the 100% transmittance count. These values along with the integration time are printed to provide hard copy of the relevant parameters for the analysis.

The routine equilibrium program will then prompt the analyst to provide the measurement parameters for the standards and samples that follow. The number of integration periods to be averaged for each measurement, the delay time before data acquisition begins, the number of standards to be measured, and the number of measurements to be averaged per standard are required. The program then sequences through each standard, flushing the system between each standard and prompting the operator for its concentration. After each standard is run, the printer prints the average absorbance, and the relative standard deviation of the measurments for each run. The program can also provide the standard deviation obtained from the integrations done for each measurement.

After the standards have been measured, the microcomputer will calculate the linear least-squares regression line and print its slope, intercept, correlation coefficient, and the standard error of the estimate. Samples are then measured, after which the concentration of the analyte in the sample is calculated from the regression line and then printed.

Rate measurements are calculated by measuring the average rate of change of absorbance over a designated measurement interval. A series of twenty-three absorbance values are obtained and fitted to a line using the linear least-squares regression. The initial and final absorbance values along with the slope and intercept are recorded on the printer. The percent uncertainty in this slope based on the 95% confidence interval using the Student's t is also printed as an indication of the quality of the fit. The absorbance values for the twenty-three points in each measurement can also be printed. The routine rate program follows a sequence similar to the equilibrium program when sequencing through a series of standards and samples.

In the dedicated mode the program required to perform the desired determination is then loaded by the operator. Standards, made up to preselected concentrations (contained in the software) are then placed in the correct order on the turntable, along with the samples. The type of determination to be performed is printed and the computer prompts the operator to input the number of samples to be assayed. Once this is input, the system is sequenced through both standards and samples automatically and no further operator interaction with the computer is needed. After all determinations have been made, the statistical analysis of the working curve is printed along with the measured values for each sample.

### Characteristics of the sampling/mixing module

Several tests were performed to evaluate the suitability of the sampling/mixing module for use in both reaction-rate and equilibrium measurements. The precision with which solution could be aliquoted, delivered, and mixed was determined by two types of tests. The first involved the measurement of the precision obtained for dilution of a dye. For this study a solution of 2,6-dichlorophenol-indophenol (DCPI) (Sigma Chemical Co., St. Louis, MO) in a 210 mg/l sodium hydrogen carbonate solution was used to give an approximate absorbance = 1 in the observation cell (10 mm pathlength) when mixed with an equal volume of sodium hydrogen carbonate solution. Table 2 shows the results obtained for a series of 10 successive cycles of the sampling/mixing module. Absorbance measurements were carried out at 520 mm which is the

**Table 2. Reproducibility of diluting a dye***

| Run | Absorbance | % RSD† |
|---|---|---|
| 1 | 1.0434 | 0.05 |
| 2 | 1.0436 | 0.03 |
| 3 | 1.0445 | 0.04 |
| 4 | 1.0439 | 0.03 |
| 5 | 1.0440 | 0.07 |
| 6 | 1.0435 | 0.02 |
| 7 | 1.0423 | 0.02 |
| 8 | 1.0436 | 0.04 |
| 9 | 1.0438 | 0.04 |
| 10 | 1.0421 | 0.04 |
| Average | 1.0435 | |

Relative standard deviation = 0.08%

* Dilution of 2,6–Dichlorophenol-indophenol with $NaHCO_3$ solution, λ = 520nm, delay time = 5 seconds, measurement time = 6 seconds

† % RSD of 10 integrations

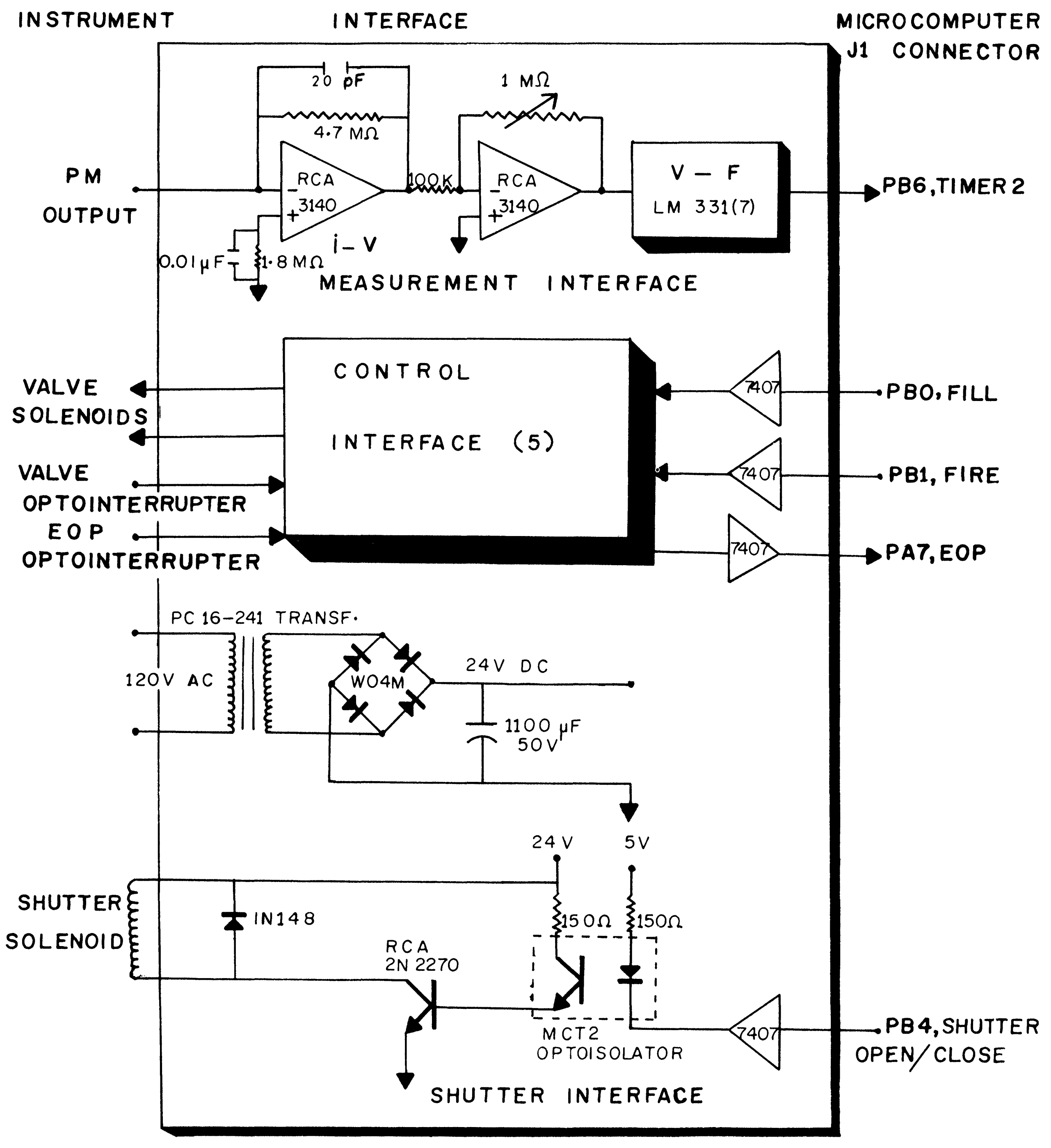

*Figure 5. Diagram of the system interface.*

isosbestic point of DCPI. The relative standard deviation was found to be 0.08% for this dilution study. A series of DCPI solutions were also mixed with equal volumes of 0.05 M oxalic acid solution and the absorbance values varying from 0.2 to 2 absorbance units were measured during a 3 second interval 30 seconds after the mixing. The results obtained are shown in Table 3.

The second test involved the molybdenum blue method for the determination of phosphorus. The methodology used was a slight modification of that used by McCracken and Malmstadt [8]. The rate of formation of the phosphomolybdenum blue is followed at 660 nm for 4 seconds after a 2 second delay time. Ten successive cycles were measured and the results in Table 4 show a relative standard deviation of 0.37% in this study. The syringes, as described previously, hold a maximum of 250 μl each. The minimum volume required to move the old solution from the cell (dead volume of mixer, flow cell and their connections) was found to be 100-250μl can be chosen by adjusting the mechanical stoppers of the automatic pipettor. Smaller volumes might be desired for expensive reagents or limited samples, but the reproducibility will be less with smaller volumes. If feasible, dilution of samples and reagents is recommended.

### Solution carry-over

An important parameter to be measured is the number of flushes needed to change from one solution to another. This is important in terms of both time and reagent conservation. To determine the number of flushes required, the sample tube was placed in a DCPI solution and the device cycled until a reproducible absorbance was obtained. After the fourth cycle the absorbance value is within one standard deviation of the final absorbance. The delivered volume per syringe was 150 μl so that 450 μl are required for flushing the system. This volume is greater than that described previously [5], but is not a problem with many procedures.

**Table 3. Results for 2,6–dichlorophenol-indophenol calibration curve***

| [DCPI] relative concentration | Absorbance | % RSD(n=5) |
|---|---|---|
| 1.00 | 0.2060 | 0.14 |
| 2.00 | 0.4185 | 0.05 |
| 3.00 | 0.6446 | 0.13 |
| 4.00 | 0.8632 | 0.18 |
| 5.00 | 1.0817 | 0.06 |
| 6.00 | 1.3001 | 0.10 |
| 7.00 | 1.5210 | 0.09 |
| 8.00 | 1.7409 | 0.11 |
| 9.00 | 1.9500 | 0.15 |

* Calibration curve: slope=0.2189, intercept=–0.0137 r=0.99997

**Table 4. Reproducibility of rate measurements for phosphorus determination**

| Run | Rate (mA/Sec) |
|---|---|
| 1 | 70.44 |
| 2 | 70.58 |
| 3 | 70.91 |
| 4 | 70.99 |
| 5 | 70.69 |
| 6 | 71.34 |
| 7 | 70.89 |
| 8 | 71.11 |
| 9 | 70.85 |
| 10 | 70.94 |
| Average | 70.87 |

Relative Standard Deviation = 0.37%

* Reduction of molybdophosphoric acid with ascorbic acid, λ = 660nm; delay time = 2 seconds; measurement time = 4 seconds

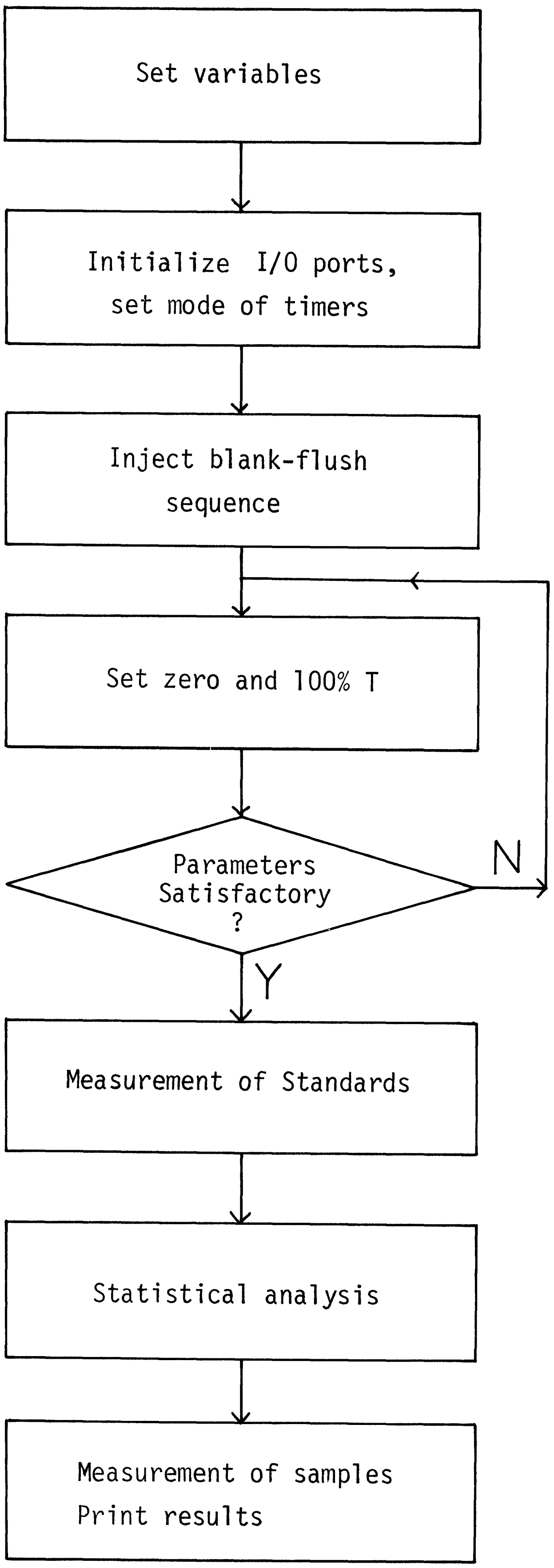

*Figure 6. Flowchart for a typical sequence in a determination using interactive routine mode program.*

### Dead time

The dead time, the time needed to fill the observation cell with a freshly mixed solution, depends on the rate the two syringes of the pipettor are moving and on the volume delivered. With a 24 psi air pressure for delivery and 200μl total volume delivered a dead time of 200 msec has been obtained. Very fast reactions with half-life time less than 200 msec can not be used for reaction rate methods.

### Chemical results

Generally to gain the fullest advantage of the rapid mixing characteristics of the stopped flow technique, fast reactions must be studied. However, most analytical methods widely used in several fields are quite inadequate for this technique because of the limitations imposed by the currently available commercial instrumentation used in their development. The majority of reaction-rate methods are slow enough to be monitored, several seconds after initiation of the reaction. Even for the slower methods however, this system can be advantageous since it can automatically provide the solution manipulations required for the reactants.

To illustrate the ability of application of this instrument in both equilibrium and reaction-rate measurements, methods for ascorbic acid, iron (III), phosphorus, creatinine and glucose have been developed and are described here.

### Ascorbic acid determination

This procedure is based on the official USP titrimetric method in which DCPI is used as titrant. A reaction-rate procedure has been reported [9, 10] which measured the rate of the DCPI-ascorbic acid reaction with a stopped-flow instrument. However, because this reaction is very rapid an equilibrium method is proposed.

*DCPI reagent* 2.907 x $10^{-4}$ M in sodium hydrogen carbonate solution (210 mg/1).

*Ascorbic acid standards* 5-40 g/l in 0.05M oxalic acid solution.

*Procedure* The sampling/mixing module is used to sample 150μl volumes of the reagent and standards and the absorbance of the reaction is measured at 520 nm during a 3 second measurement interval, 30 seconds after mixing of the reactants.

*Results and discussion* Results from a set of working curve standards are shown in Table 5. The working curve is linear with a correlation coefficient of 0.9994 and a relative standard deviation of 0.1 to 0.7%.

### Iron (III) determination

Iron (III) can be determined by measuring the absorbance after the reaction with ammonium thiocyanate at 480 nm, in acid solution.

*Iron (III) standard solutions* 2.00-20.00 ppm Fe(III) prepared from a Fe $(C1O_4)_3$ stock solution in 1.0M $HNO_3$.

*Ammonium thiocyanate* Aqueous solution, 1%.

*Procedure* Iron (III) standards are mixed with the reagent using the sampling/mixing module and the absorbance at 480 nm during a 6 second measurement interval, 10 seconds after the mixing of the reactants.

*Results and discussion* Results from a set of working curve standards are shown in Table 6 and give a correlation coefficient of 0.99996 and relative standard deviations of 0.1 to 1.0%

**Table 5. Results used for determination of ascorbic acid***

| Ascorbic acid g/l | Absorbance | %RSD |
|---|---|---|
| 5.00 | 0.9122 | 0.14 |
| 10.00 | 0.8052 | 0.29 |
| 20.00 | 0.5760 | 0.40 |
| 30.00 | 0.3336 | 0.33 |
| 40.00 | 0.0853 | 0.72 |

* Working curve: slope = –02366, intercept = 1.039, r = 0.9994

† Average of 5 determinations on a single sample

### Phosphorus determination

This method is based on the rate of reduction of molybdophosphoric acid with ascorbic acid. The formation of the phosphomolybdenum blue product is followed at 660 nm for 4 seconds after a 2 second delay time [8].

Phosphorus standards: The phosphorus standards were made by diluting a stock solution of 100 ppm phosphorus prepared with 0.4393 g of dipotassium hydrogen phosphate. This was mixed in a 1 litre flask to which 34.8 ml. of concentrated sulphuric acid was added before diluting to bulk.

*Ascorbic acid reagent* This reagent was prepared daily with 0.53 g of ascorbic acid and 0.656 g of sodium hydroxide dissolved in 100 ml of demineralized water.

*Molybdenum reagent* The molybdenum reagent (0.5M) was prepared a day before its use. Sodium molybdate dihydrate (1.209 g) was added to 100 ml of 0.1M sulphuric acid.

*Procedure* The phosphorus stock solution was diluted to its desired concentration and then added in a 1:4 ratio to the ascorbic acid reagent to provide standards ranging from 4-20 ppm phosphorus. The standards are injected along with the molybdenum reagent with the sampling/mixing module and the change in absorbance at 660 nm is measured for 4 seconds after a 2 second delay time.

*Results and discussion* The results from a set of working curve standards are shown in Table 7. These results in a correlation coefficient of 0.9998 and relative standard deviations ranging from 0.4 to 0.9%.

### Creatinine determination

This method is based on the rate at which creatinine forms a colour complex with alkaline picrate (Jaffe reaction) measured at 510 nm.

*Picric acid solution* 10.1 g of picric acid crystals are dissolved in 1 litre of distilled water.

Sodium Hydroxide 0.75M solution.

*Alkaline picrate working solution* Equal volumes of picric acid and sodium hydroxide are mixed just before use.

*Standard creatinine solutions* 1.0-5.0 mg/dl (Sigma Creatinine Standard Solutions, series 925.)

*Procedure* The Creatinine standards are injected along with the alkaline picrate working solution with the sampling/mixing module and the rate of the reaction is measured for 15 seconds after a 15 second delay time.

*Results and discussion* The results from a set of working curve standards are shown in Table 8. As shown, a correlation coefficient of 0.9994 and relative standard deviations of 0.2 to 0.6 have been obtained.

**Table 6. Results used for determination of iron (III)***

| Fe(III), ppm | Absorbance † | %RSD |
|---|---|---|
| 2.00 | 0.1055 | 1.0 |
| 6.00 | 0.3170 | 0.3 |
| 12.00 | 0.6355 | 0.1 |
| 16.00 | 0.8477 | 0.4 |
| 20.00 | 1.0526 | 0.2 |

* Working curve: slope = 0.0527, intercept = 0.0011, r = 0.99996

† Average of 5 determinations

**Table 7. Results for determination of phosphorus***

| P, ppm | ΔA (ma/sec)† | %RSD |
|---|---|---|
| 4.00 | 24.91 | 0.53 |
| 8.00 | 47.53 | 0.91 |
| 12.00 | 70.87 | 0.37 |
| 16.00 | 94.26 | 0.61 |
| 20.00 | 118.31 | 0.91 |

* Working curve: slope = 5.838, intercept 1.117, r = 0.9998

† Average of 5 measurements

Table 8. Results used for determination of creatinine*

| Creatinine (mg/dL) | Rate (mA/sec)† | %RSD |
|---|---|---|
| 1.00 | 3.99 | 0.18 |
| 2.00 | 7.32 | 0.48 |
| 3.00 | 10.17 | 0.58 |
| 4.00 | 13.16 | 0.47 |
| 5.00 | 16.30 | 0.45 |

* Working curve: slope = 3.046, intercept 1.052, r = 0.9994

† Average of 5 determinations

Table 9. Results used for determination of glucose*

| Glucose (mg/dL) | Rate (mA/sec)† | %RSD |
|---|---|---|
| 1.92 | 2.13 | 1.0 |
| 9.60 | 6.53 | 0.31 |
| 14.40 | 9.50 | 0.26 |
| 19.20 | 12.06 | 0.36 |
| 32.00 | 19.57 | 0.24 |

* Working curve: slope = 0.5794, intercept 1.022, r = 0.9997

† Average of 5 measurements

Table 10. Determination of glucose in control sera

| Control sera | Manufacturers stated value mg/dL | Value found mg/dL |
|---|---|---|
| Monitrol* I | 94 ± 1.8 | 90 ± 1.1 |
| Monitrol II | 224 ± 3.6 | 224 ± 2.1 |
| Monitrol II | 228 ± 4.2 | 227 ± 1.7 |

* Dade Division, American Hospital supply, Miami FL 33152

Table 11. Routine analysis rates

| Determination | Analysis rate (samples/hour) | Delay time (sec) | Meas. time (sec) |
|---|---|---|---|
| Ascorbic acid* | 90 | 30 | 0.6 |
| Iron (III)* | 183 | 10 | 0.6 |
| Phosphorus† | 240 | 2 | 4 |
| Creatinine† | 92 | 15 | 15 |
| Glucose† | 109 | 12 | 12 |

*: Equilibrium method, †: Reaction-rate method

**Glucose determination**

Glucose is oxidized to gluconolactone by glucose oxidase and in the presence of oxygen and water, gluconolactone is further converted to gluconic acid and hydrogen peroxide. The hydrogen peroxide reacts with p-diphenylamine sulphonate to form a chromagen, which absorbs at 470 nm in a reaction rate method.

Citrate buffer pH 5.5, 0.1M.

*Glucose reagent* 120 mg of glucose oxidase (Sigma Chem. Co. Pro. No. 6125), 77 mg of horseradish peroxidase (Sigma) and 180 mg of sodium p-diphenylamine sulfonate (Frederick Smith) are dissolved in 100 ml of citrate buffer. This reagent is kept in the refrigerator when not used.

*Glucose standard solutions* 2-32 mg/dl are prepared by appropriate dilution of a stock glucose solution.

*Procedure* Glucose standards are injected along with the glucose reagent, and the reaction rate is monitored for a 12 second measurement time 12 second after mixing. Serum samples are treated similarly after a 2:25 dilution with distilled water.

*Results and discussion* The results from a set of working curve standards are shown in Table 9. A correlation coefficient of 0.9997 and relative standard deviations of 0.2 - 1.0% have been obtained. The method was applied on three control serums (Dade, Moni-trol) and the results obtained are shown in Table 10.

### Conclusion

From the above typical examples it is clear that this compact stopped-flow autoanalyzer can be used for equilibrium or reaction rate methods with accurate and precise results. For equilibruim methods the in-run precision for 10 integrations is always better than 0.1% so that only one integration (0.6 sec measurement time) can be used. The between runs precision for both methods is also better than 1% so for a routine analysis of a large number of samples one measurement per sample gives reliable results.

Assuming four flushes to change from one solution to another (flush cycle time about 1.5 sec), one measurement per sample, one sec for the turntable position increment, and 0.5 sec for the computer calculation and printing time the analysis rates for the aforementioned methods shown in Table 11 can be obtained. The total sample of reagent volume consumed is 750 μl.

ACKNOWLEDGEMENTS

The work was supported in part by a grant from HEW NIGMS 21984.

REFERENCES

[1] Javier, A.C., Crouch, S.R. and Malmstadt, H.V., *Analytical Chemistry,* 1969, **41**, 239

[2] Malmstadt, H.V., Cordos, E.A. and Delaney, C.J., *Analytical Chemistry,* 1972, **44**(12), 26A.

[3] O'Keefe, K.R. and Malmstadt, H.V. *Analytical Chemistry,* 1975, **47**, 707.

[4] Krottinger, D.L., McCracken, M.S. and Malmstadt, H.V., *Talanta,* 1979, **26**, 549.

[5] Krottinger, D.L., McCracken, M.S., and Malmstadt, H.V., *American Laboratory,* 1979, 9(3), 51.

[6] Krottinger, D.L., McCracken, M.S., and Malmstadt, H.V., *The Journal of Automatic Chemistry,* 1978, **1**(1), 15.

[7] Linear Data Book. p. 8-103. National Semiconductor Corporation, 1978.

[8] McCracken, M.S. and Malmstadt, H.V., *Talanta,* 1979, **26**, 467.

[9] Karayannis, M.I., *Analytical Chimica Acta,* 1975, **76**, 121.

[10] Karayannis, M.I., *Talanta,* 1976, **23**, 27.

